# Comparison of major complications in children after laparoscopy-assisted gastrostomy and percutaneous endoscopic gastrostomy placement: a meta-analysis

**DOI:** 10.1007/s00383-018-4358-6

**Published:** 2018-10-05

**Authors:** Filip Sandberg, Margrét Brands Viktorsdóttir, Martin Salö, Pernilla Stenström, Einar Arnbjörnsson

**Affiliations:** 1 Department of Pediatric Surgery, Skåne University Hospital, 221 85 Lund, Sweden; 2 Department of Clinical Sciences, Pediatrics, Lund University, Lund, Sweden

**Keywords:** Meta-analyses, Gastrostomy in children, Complications, Laparoscopy-assisted gastrostomy, Percutaneous endoscopic gastrostomy

## Abstract

**Purpose:**

A meta-analysis was performed to compare the rates of the major complications associated with two gastrostomy tube placement techniques in a pediatric population: laparoscopy-assisted gastrostomy (LAG) and percutaneous endoscopic gastrostomy (PEG).

**Methods:**

The PubMed electronic database was queried for comparative studies of the two insertion techniques. The Newcastle–Ottawa scale (NOS) was used for the assessment of the quality and risk of bias in the included studies. The main outcome measure was the frequency of major complications defined as the need for reoperation within 30 days or death. RevMan 5.3, was used, with a *p* < 0.05 indicating statistical significance.

**Results:**

Eight studies including 1550 patients met the inclusion criteria. The risk for major complications was higher in PEG than in LAG 3.86 (95% confidence interval 1.90–7.81; *p* < 0.0002). The number needed to treat to reduce one major complication by performing LAG instead of PEG was 23. There were no randomized-controlled trials. Overall, the quality of the included studies was determined to be unsatisfactory.

**Conclusions:**

PEG placement was associated with a significantly higher risk of major complications compared to LAG placement. Therefore, LAG should be the preferred method for gastrostomy tube placement in children.

## Introduction

Enteral feeding using a gastrostomy tube in children should be considered if adequate nutritional intake will not be adequate in 2–3 weeks’ time [1]. Currently, gastrostomy placement is one of the most common surgical interventions among children [2] and the spectrum of diagnosis with indications for gastrostomy is broad including neurological, metabolic, cardiac, pulmonary, and renal disorders.

First described in 1894, gastrostomy was initially performed by the conventional open surgery [3]. The less invasive percutaneous endoscopic gastrostomy (PEG) tube placement, introduced in 1980 [4], is still widely used in pediatric and adult patients. After 1990, laparoscopy and other video-assisted techniques have been used increasingly in abdominal and thoracic surgical interventions [5], and Andersson et al. [6] were one of the first to describe the laparoscopy-assisted gastrostomy (LAG) tube placement method in 1997. This method has later been modified and developed [7]. However, the reports of serious complications associated with the PEG technique in children have raised concerns regarding the safety and utility of both the two approaches [8, 9] in pediatric populations.

This meta-analysis aimed to compare the rates of major complications in children after gastrostomy placement using the PEG and LAG techniques.

## Methods

### Search strategies

The meta-analysis was conducted following the PRISMA guidelines [10]. All the literature published from 1990 until January 2018 was searched using the PubMed database. First, the search was performed using the search function “ALL FIELDS”. Abbreviations and several synonyms were included for different intervention techniques, including PEG, percutaneous endoscopic gastrostomy, laparoscopy-assisted gastrostomy, video-assisted gastrostomy, laparoscopic gastrostomy, and laparoscopic assisted gastrostomy. The search query included “complications”. Filters were set for articles published in English and included the age groups: infants, children, and adolescents.

First, all the abstracts were screened, and all the studies examining major complications after the PEG and LAG tube placement as an outcome were considered to meet the inclusion criteria. Then, the full articles were retrieved. All the eligible abstracts and articles were assessed independently by FS and EA for inclusion in the meta-analysis.

### Inclusions criteria

Included were all comparative studies on children younger than 18 years reporting major complications associated with two gastrostomy tube placement techniques: LAG and PEG.

### Exclusion criteria

Excluded were all non-original articles, those with cohorts smaller than ten patients, those with a ratio of greater than 10:1 between the two intervention techniques, and those lacking presentation of complications. Studies including individuals who underwent fundoplication in addition to the gastrostomy were excluded if it was not possible to differentiate those individuals from those undergoing gastrostomy tube placements alone. Studies reporting overlapping data and previously published articles were excluded. To reduce the risk of modification of methods that might influence the rate of complications, studies with time interval spanning greater than 15 years were also excluded.

### Complications

In the current study, major complications were defined according to Clavien–Dindo grade 3b complications [11] requiring reoperation within 30 days and included organ damage, excessive intraabdominal leakage, and fistula formation. Minor complications, such as granuloma, infections, vomiting, and pain, were not considered as major complications even if they resulted in reoperation within 30 days in the current study.

### Data extraction

The data extracted from the included articles were study characteristics including authors, publication year, sample size, time span, surgical technique (PEG or LAG), follow-up period, and patient characteristics including sex, age at surgery, weight, diagnosis, and indication for gastrostomy tube placement. Specific information on postoperative complications was collected and analyzed. To avoid duplicate entry of cases, the institution from which data were collected was also noted. Furthermore, in articles not delineating the time from the first operation to the complication, the need for intervention within 30 days had to be deduced. This was done as the great majority of complications were described to happen in time close to the surgery and that our chosen time interval of 30 days is long and suspected to include most postoperative complications for this type of surgery. However, in cases of uncertainty, events were not included as major complications.

### Quality assessment

The Newcastle–Ottawa scale (NOS) was used for an independent assessment of quality of the studies [12]. The scale constitutes three different aspects of quality assessment, including selection, comparability, and outcome. Relevant measurements were awarded with 0–9 (best = 9) stars according to the NOS model. Studies with a low, moderate, and high risks of bias were allocated 9–7, 4–6, and 0–3 overall stars, respectively. In the outcome category, studies earned stars if secure reports were available for the analyses of outcomes, (1 star), if the follow-up was greater than 30 days (1 star), and if not more than 10% of the study individuals were lost to follow-up (1 star). To achieve these stars, the study had to be designed to fulfill these requirements.

### Statistical analysis

The Mantel–Haenszel method was used to calculate pooled odds ratios (ORs) [13]. Dichotomous variables were analyzed by estimating ORs with 95% confidence intervals (CIs), and continuous variables were analyzed using weighted mean differences with 95% CIs. *p* values < 0.05 were considered statistically significant. The RevMan statistical package version 5.3 was used to conduct the meta-analysis [14].

## Results

### Literature search

**Fig. 1:**
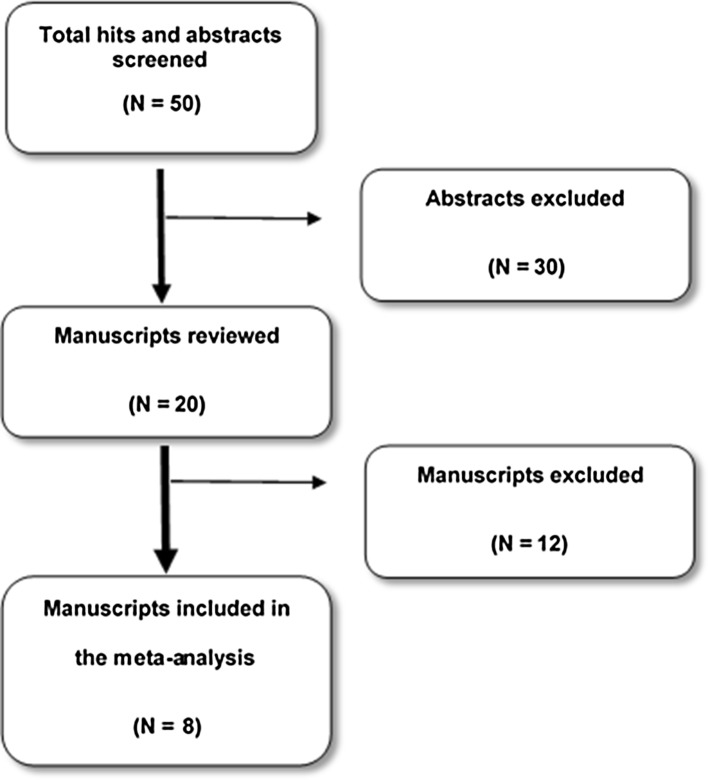
Flowchart of the search process to locate articles to compare major complications after gastrostomy insertion in children with the percutaneous endoscopic and laparoscopy-assisted techniques

A total of 50 abstracts were screened, of which 20 studies met the eligibility criterion. The full texts were read, and eight observational studies were deemed to fulfill the criteria to be included in the final meta-analysis [15–22] (Fig. 1). None of the analyzed studies were randomized-controlled trials.

### Study characteristics

A total of 1550 patients (range 69–346 patients/study) could be included in the meta-analysis. Of these, 866 (56%) underwent PEG and 684 (44%) underwent LAG. Some local variations in the surgical methods were noted during the data extraction, although the general principle of the methods was equal and allowed for comparison. Data on sex, age at surgery, weight, diagnosis, and indication for gastrostomy tube placement were provided in very few studies and, therefore, the information was not included in the current analysis. Table 1 presents a summary of the characteristics of the included studies.

**Table 1:** Characteristics and Newcastle–Ottawa Scale stars of eight studies included in the meta-analysis to compare major complications in children after gastrostomy insertion using percutaneous endoscopy gastrostomy and laparoscopy-assisted gastrostomy

References	Study period	*N* (% total)	PEG (% total)	LAG (% total)	NOS star (max, 9 stars)
Akay et al. [15]	2004–2008	238 (15.3%)	134 (15.5%)	104 (15.2%)	6^a,b,d^
Landisch et al. [16]	2011–2015	183 (11.8%)	78 (9.0%)	105 (15.3%)	8^b^
Liu et al. [17]	1998–2010	346 (22.3%)	86 (9.9%)	260 (38.0%)	6^a,b,c^
Merli et al. [18]	2004–2015	69 (4.5%)	37 (4.3%)	32 (4.7%)	6^a,b,c^
Petrosyan et al. [19]	2009–2014	225 (14.5%)	150 (17.3%)	75 (11.0%)	7^b,c^
Sulkowski et al. [20]	2010–2012	206 (13.3%)	181 (20.9%)	25 (3.7%)	7^a,b^
Wragg et al. [21]	2006–2009	164 (10.6%)	107 (12.4%)	57 (8.3%)	6^a,b,c^
Zamakhshary et al. [22]	2002–2003	119 (7.7%)	93 (10.7%)	26 (3.8%)	6^a,b,c^

*N* number of subjects, *NOS* Newcastle–Ottawa Scale, *PEG* percutaneous endoscopic gastrostomy, *LAG* laparoscopy-assisted gastrostomy

^a^Reduction in NOS star because of > 5 years in age interval among included patients

^b^Reduction in NOS star because of unequal comorbidities between the PEG and LAG groups

^c^Reduction in NOS star because of a follow-up period < 30 day or not specified

^d^Reduction in NOS star, because > 10% of patients were lost to follow-up

### Major complications

**Fig. 2:**
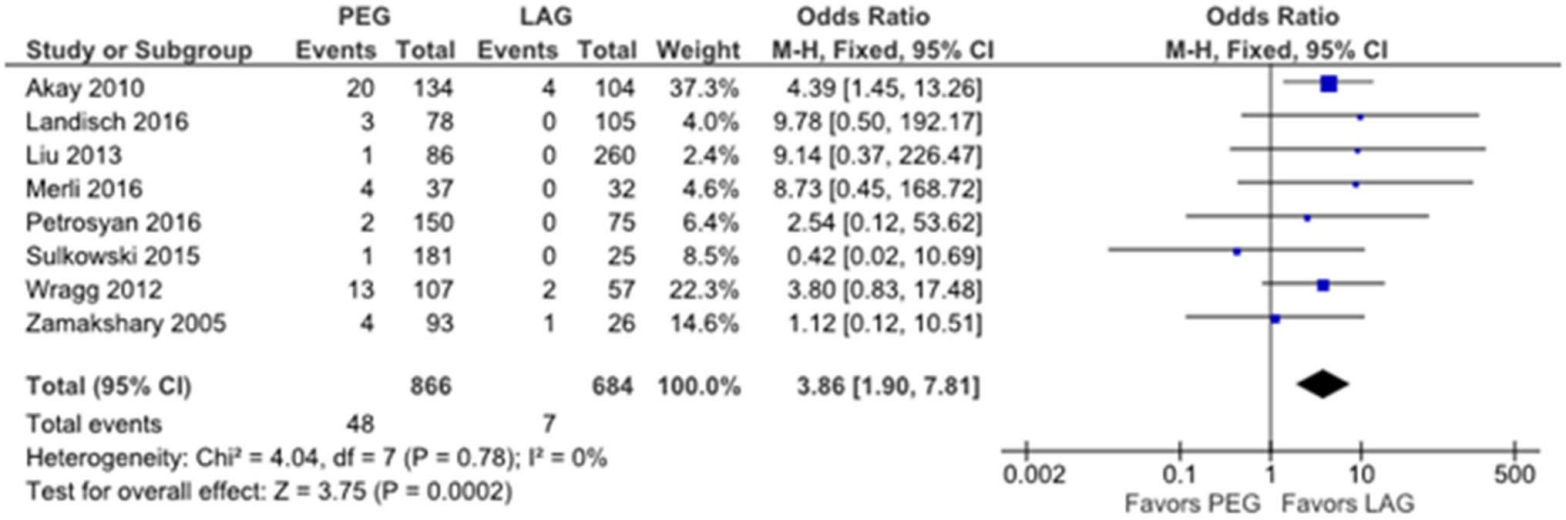
Forest plot showing major complications in children after laparoscopy-assisted gastrostomy versus percutaneous endoscopic gastrostomy tube insertion. *PEG* percutaneous endoscopic gastrostomy, *LAG* laparoscopy-assisted gastrostomy, *M–H* Mantel–Haenszel, *CI* confidence interval

**Fig. 3:**
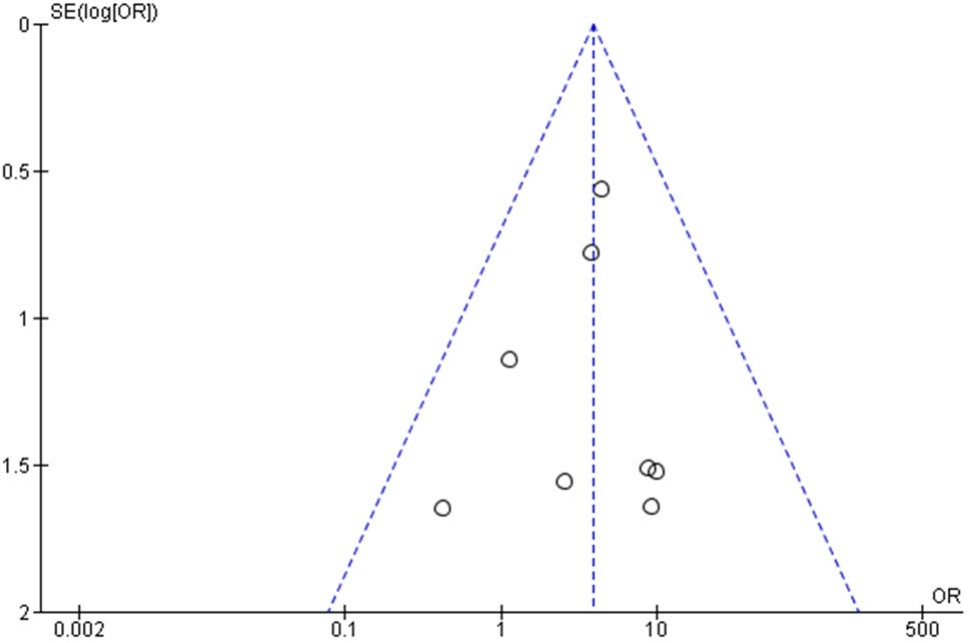
Funnel plot to assess bias in reports on major complications in children undergoing laparoscopy-assisted gastrostomy and percutaneous endoscopic gastrostomy for tube insertion. The *X* axis shows odds ratio, OR, and the *Y* axis shows the inverse standard error, the log of the OR

Overall, 55 out of a total of 1550 (3.6%) patients exhibited a major complication requiring reoperation within 30 days. Specifically, major complications occurred in 48 of 886 (5.4%) and 7 of 684 (1.0%) patients who underwent PEG and LAG tube placement procedures, respectively. All the studies reported lower frequencies of major complications in children who underwent the LAG compared to those who underwent the PEG. The overall pooled OR was 3.86 (95% CI 1.90–7.81, *p* = 0.0002) favoring LAG (Fig. 2). Figure 3 presents a funnel plot to assess bias in the included studies, showing symmetry and suggesting only minor bias.

The most common complication in the PEG group was of mechanical characteristics such as accidental tube dislodgement, intraperitoneal tube leakage, and failed tube placement, reported in 44% (21/48) of the patients with a major complication. In the LAG group, the most common major complication was the appearance of fistulas, reported in 57% (4/7) of the patients with a major complication. In one publication only [15], three gastro cutaneous fistulas were described in the LAG group and five in the PEG group, respectively. All these happened within the 30 days period studied. These complications would not influence the statistically significant difference between the LAG and PEG groups. Table 2 presents the complications associated with the specific tube placement technique. The number needed to treat to reduce one major complication by preferring an LAG procedure instead of PEG tube placement approach was 23.

**Table 2:** List of major complications in children needing reoperation within 30 days after gastrostomy insertion using one of the two techniques

References	*N*	*N*	Major complications *N* (%)		Fistula formation *N*		Organ injury *N*		Pneumoperitoneum *N*		Mechanical *N*		Other *N*	
	PEG (*P*)	LAG (*L*)	*P*	*L*	*P*	*L*	*P*	*L*	*P*	*L*	*P*	*L*	*P*	*L*
Akay et al. [15]	134	104	20 (15%)	4 (3.8%)	6	4	1	0	2	0	11	0	0	0
Landisch et al. [16]	78	105	3 (3.8%)	0 (0%)	3	0	0	0	0	0	0	0	0	0
Liu et al. [17]	86	260	1 (1.2%)	0 (0%)	1	0	0	0	0	0	0	0	0	0
Merli et al. [18]	37	32	4 (10.8%)	0 (0%)	0	0	0	0	0	0	0	0	4	0
Petrosyan et al. [19]	150	75	2 (1.3%)	0 (0%)	2	0	0	0	0	0	0	0	0	0
Sulkowski et al. [20]	181	25	1 (0.6%)	0 (0%)	0	0	0	0	0	0	0	0	1	0
Wragg et al. [21]	107	57	13 (12%)	2 (3.5%)	0	0	3	1	1	0	9	1	0	0
Zamakhshary et al. [22]	93	26	4 (4.3%)	1 (3.8%)	0	0	3	0	0	0	1	1	0	0

*N* number of subjects; *P* percutaneous endoscopic gastrostomy; *L* laparoscopy-assisted gastrostomy; *Mechanical* tube dislodgement, intraperitoneal tube leakage, failed tube placement

### Study quality

The quality assessment of the studies using the NOS for cohort studies [12] revealed that, in general, the included studies had a moderate risk of bias. Five studies were given six stars, corresponding to a moderate risk of bias, and three studies were given seven or eight stars, corresponding to a low risk of bias. The most common methodological weakness among all studies was inadequate selection of patients in both the PEG and the LAG groups, because all were retrospective cohort studies with no randomization. Demographic features and follow-up descriptions varied substantially between the studies. Five studies did not adequately report the duration of follow-up of more than 30 days. One study [15] lost greater than 10% of the patients to follow-up. Thus, only three studies reported reliable follow-up data. Table 1 presents the NOS scores of the studies included in the meta-analysis.

## Discussion

The findings in the current meta-analysis demonstrate a wide spectrum in the frequencies of major complications among the two most commonly used methods, LAG and PEG, for gastrostomy tube placement in the pediatric population. The LAG technique was associated with fewer major complications with a rate of 1% compared to the major complication rate of 5.4% in the PEG technique.

In a previous meta-analysis on gastrostomy placement in children, using death or reoperations within the first postoperative year as the end points, Baker et al. [2] have also found that the LAG technique was more favorable compared with the PEG technique, with a calculated OR of 0.29 and a 95% CI of 0.17–0.51 (*p* = 0.0001), and 45 cases were needed for treatment to reduce one major complication by abandoning PEG placement. Another support of LAG prior PEG regarding complications has also been shown in a previous review of complications after gastrostomy placements [23] and a recent meta-analysis with fewer studies [24].

Table 3 is a discussion table that summarizes the advantages and disadvantages of the two techniques. Due to the characteristics of the described complications in the literature, the most likely reason for the better safety of the LAG technique compared with the PEG technique is the advantage of direct visualization of the peritoneal cavity during the establishment of gastrostomy, avoiding the blind puncture through the abdominal cavity that is necessary during tube placement using the PEG technique. Visualization of the ventricle is of specific concern in children, who anatomically have short distances and narrower abdominal cavity than adults. One other technical advantage of operating under direct visualization is the opportunity to choose a precise site for ventricular incision, with a minimization of tension.

**Table 3:** Schematic comparison of the laparoscopy-assisted gastrostomy and the percutaneous endoscopic gastrostomy techniques in pediatric patients requiring gastrostomy tube placement

Parameters compared	LAG	PEG
Operation under general anesthesia	Yes	Yes
Blind puncture through the abdominal cavity^a^	No	Yes
Adhesion of the stomach to the abdominal wall	Suturing of the stomach to the abdominal wall	Reliance on wound healing and granulation tissue formation
Invasiveness, number of transabdominal wall wounds	2	1
Pneumoperitoneum	Yes	No
Infection, number of wounds	2	1
Cosmetics, number of scars on the abdominal wall	2	1
Repeat general anesthesia needed for a change in the gastrostomy button	No	Yes

*PEG* percutaneous endoscopic gastrostomy, *LAG* laparoscopy-assisted gastrostomy

^a^The reason for the abdominal complications

Then, one advantage of the LAG technique is the avoidance of serious complications such as unintended damage to abdominal organs as placement of the gastrostomy in colon or in the liver, as well as undiscovered bleeding. This is because one disadvantage with PEG is the inflation of the stomach prior to puncturing, because it might cause a rotation of the gastro-colic omentum and transverse colon, placing it anterior to the stomach and, thus, increasing the risk of inadvertently puncturing the colon [8, 15]. A literature review including publications from 1995 until November 2009 [8] revealed a significant difference in the complication rates between the LAG and PEG techniques. In that study, serious complications such as gastro-colic fistula formation were reported in approximately 2–4% of the gastrostomy insertions using the PEG technique.

However, the LAG technique is not suitable for all patients, and the risks associated with each technique depend on the patients’ background and the underlying medical conditions. For example, the creation of pneumoperitoneum during laparoscopic surgery may be unsafe in some patients with underlying respiratory disease. In addition, a history of prior upper abdominal surgery may complicate the operative course [23].

All the studies included in the current meta-analysis reported a longer operative time in LAG and, consequently, a longer anesthesia time for the LAG tube placement, which might be a disadvantage that must be countered with the greater risk of requiring a second surgical procedure because of a possible major complication encountered using the PEG technique. In addition, most pediatric patients are provided with a gastrostomy button, directly placed as part of the LAG tube placement which the PEG technique does not always allow. However, there are other types of PEGs done which do not require general anesthesia for a future exchange. Subsequently, some children undergoing the PEG tube placement must undergo repeated anesthesia for button placement, increasing the total time under general anesthesia to close to or even longer than that of the LAG tube placement.

Another risk moment is during the healing phase of the gastrostomy. While the PEG technique relies on adhesions to occur spontaneously, the surgeon can manually suture the stomach to the abdominal wall during the laparoscopic surgery, which reduces the number of dislodged tubes that must be reinserted and is, consequently, associated with a lower number of dislodgement complications [15–22]. However, there are now techniques described for PEG placement where T-fasteners are used in lieu of sutures to pexy the stomach to the abdominal wall prior to placement of the gastrostomy tube. These techniques were not used in the papers included in this meta-analysis. One other technical advantage of operating under direct visualization is the opportunity to choose a precise site for ventricular incision, with a minimization of tension.

Although there are many advantages to the LAG technique, the risk of future complications from adhesions and subsequent ileus due to the extra ports needed for the laparoscopic instruments remains to be evaluated.

Many children in need of a gastrostomy tube have substantial comorbidities, and every intervention constitutes a potential risk, especially if general anesthesia is needed. In addition, age at the time of surgery and comorbidities varied among the included studies. None of the eight included studies received an extra NOS star in our quality assessment for the adjustment of comorbidities, although several of the studies indicated that the groups coincidentally had similar comorbidities. For example, one study [21] showed that the comorbidities were worse among children who underwent the LAG tube placement. One might anticipate a greater risk of complications among younger patients, since their anatomy increases the risk of organ injury, but we were unable to correct the incidence of complications for age.

Importantly, individual past medical histories and comorbidities of patients should be considered in planning for future surgical interventions. Although the current meta-analysis revealed a lower complication rate associated with the LAG technique, the same might not be true for patients with the previous upper abdominal surgeries. In addition, some patients may be better served by two separate, shorter periods of general anesthesia, rendering the LAG technique disadvantageous.

The current meta-analysis has several limitations. First, there were no randomized-controlled trials in the meta-analysis, which constituted a bias. All the studies were small with a retrospective study design, which are prone to selection biases and may result in uneven distribution of confounding factors. The impact of the two studies [15, 21] with the most reported complications and the large difference in patient inclusion numbers between PEG /LAG within several studies that were included results in selection bias.

Several patient characteristics, including comorbidities, age, and sex, were heterogeneous between the treatment groups in many of the studies, which might have influenced the surgical outcomes. In addition, because not all articles presented the exact time from intervention to complication, we had to make assumptions when collecting data and delineating complications. This affected the objectivity in data collection.

Significant sources of bias in the current meta-analysis were the variations in the management of complications among different medical centers which might constitute a bias because of a difference in the definition of complications. Furthermore, the reporting of complications varied substantially among the studies evaluated in our search. Two of the studies included in the metanalysis [15, 21], Table 2, have a significant influence on the data given the small sample size of eight articles only. These reports had a higher rate of complications than the other papers and weighed heavily on the data in terms of swaying the conclusions about the safety of PEG tubes. However, we believe that we conducted our comparisons as accurately as possible.

The results of the current meta-analysis can be utilized in clinical practice as a foundation for planning gastrostomy tube placement in pediatric patients. Despite the various limitations, the results strongly favor the LAG technique, rendering a randomized-controlled trial unlikely to reach a different conclusion. In addition, given that numerous studies already favor the LAG technique, randomized-controlled trials comparing these two methods are ethically problematic.

## Conclusions

Our meta-analysis of the literature indicates that LAG tube placement is associated with a lower incidence of major postoperative complications. Randomized studies including patients at similar ages and with similar comorbidities are necessary to secure the result.
